# Exploration of Factors Affecting Post-Secondary Students’ Stress and Academic Success: Application of the Socio-Ecological Model for Health Promotion

**DOI:** 10.3390/ijerph18073779

**Published:** 2021-04-05

**Authors:** Konrad T. Lisnyj, David L. Pearl, Jennifer E. McWhirter, Andrew Papadopoulos

**Affiliations:** Department of Population Medicine, University of Guelph, 50 Stone Road East, Guelph, ON N1G 2W1, Canada; dpearl@uoguelph.ca (D.L.P.); j.mcwhirter@uoguelph.ca (J.E.M.); apapadop@uoguelph.ca (A.P.)

**Keywords:** stress, undergraduate students, Canada, post-secondary students, academic success, socio-ecological model, qualitative research, student wellness

## Abstract

Overview: There has been an increase in the frequency and severity of stress experienced by Canadian post-secondary students, which has adverse implications on their academic success. This work applied the socio-ecological model for health promotion to explore the contextual factors that influence this relationship at the individual, interpersonal, institutional, community, and public policy levels. Methods: Using a qualitative, phenomenological approach, we conducted 38 semi-structured interviews with undergraduate students and on-campus staff who provide services to this population at a post-secondary institution in Southwestern Ontario, Canada. Thematic analysis inductively identified overarching themes among participants’ perspectives. Results: Several positive and negative factors were identified at each socio-ecological model level, demonstrating the complex interplay of demographic, psychological, emotional, social, physical, and academic factors impacting students’ academic stress. Conclusions: A lack of communication and knowledge seems to underlie many factors, highlighting the need to strengthen communication strategies to promote awareness, accessibility, and availability of services and programs on campus. Results also pointed to focusing on proactive, resilience-focused, upstream mental health promotion efforts at post-secondary institutions to reduce stress and improve academic success. This knowledge can help Canadian campuses better address students’ needs.

## 1. Introduction

Canadian post-secondary students experiencing excessive stress continues to be a severe public health issue. The most recent 2019 National College Health Assessment-II (NCHA-II) survey conducted by the American College Health Association (ACHA) on a nationwide sample of Canadian post-secondary students (*n* = 55,284) found that 60.8% (95% confidence interval (CI): 60.4–61.2; CI estimated by Lisnyj et al.) of respondents reported above average stress within the past year [1], up from 57.5% (*n* = 34,039) (95% CI: 57.0–58.0; CI estimated by Lisnyj et al.) in the 2013 iteration of this same survey [2]. Post-secondary students are more vulnerable than non-post-secondary students to experiencing excess stress [3]. Post-secondary students are particularly vulnerable to experiencing stress due to their competing personal, financial, social, academic, and career-related demands, combined with them traditionally pursuing higher education during their “emerging adulthood” stage of life [4,5]. Extensive research has documented the adverse implications of stress on this population’s physical, mental, emotional, and behavioural health outcomes (e.g., [6,7,8,9]). Several researchers have also noted the negative effects of extreme stress levels on post-secondary students’ academic outcomes, including declining academic performance, disengagement, dissatisfaction, and attrition [10,11,12,13]. Scholars (e.g., [14,15]) have argued that stress literature often encompasses quantitative research and explicitly state the need for “qualitative methods to identify overlooked stressors, nuances in theory, and the importance of context” [14] (p. 276). In turn, this can help identify opportunities to mitigate students’ stress affecting their lives.

Canadian post-secondary institutions are increasingly committed to enhancing students’ mental health and academic success through services and programming [16,17,18,19]. However, Linden and Stuart’s [20] work argues that there is a limited understanding of the specific evolving sources of stress experienced by Canadian post-secondary students. Students are often excluded from institutional needs assessments, making it difficult to determine if the services offered on campus truly align with students’ needs. This information is required to inform upstream services and resources targeting effective mental health promotion (e.g., stress reduction) on campuses [20]. An additional notable gap among existing institutional health education programs and services is a lack of theoretical or conceptual underpinnings [11]. Glanz, Rimer, and Viswanath [21] state that when theory is integrated into health promotion practice, their dynamic interaction helps establish effective and sustainable interventions. In particular, interventions and systems integrating the socio-ecological model for health promotion [22] have been found to improve mental health outcomes (e.g., [23,24,25,26]).

The socio-ecological model for health promotion [22] posits five levels of factors influence individuals’ behaviours: intrapersonal, interpersonal, institutional, community, and public policy. Intrapersonal factors comprise individuals’ own characteristics, including their knowledge, attitudes, skills, and behaviours [22]. Interpersonal factors concern one’s formal and informal social networks and relationships [22]. At the institutional level, organizational characteristics, such as operating rules and regulations, are considered [22]. Community factors include relationships among several actors within a defined boundary [22]. Public policy factors exist at the most macro level and are used to examine the influence of local, national, and global laws and policies on individuals’ behaviours [22]. Although this model emerged over three decades ago, it is underused on post-secondary campuses, particularly surrounding mental health promotion. This multi-faceted model can help administrators identify and understand the multitude of factors contributing to student stress, especially as it relates to their academic success.

In this exploratory study, we applied the socio-ecological model for health promotion to understand how factors at each level influence undergraduate students’ stress levels and academic success at a post-secondary institution in Southwestern Ontario, Canada. We were additionally interested in how students’ perceptions of these factors compare and contrast to the views of other on-campus stakeholders who provide services for this population. Findings from this needs assessment research may help to identify areas for post-secondary institutions to better target their efforts to reduce student stress and optimize their academic wellness.

## 2. Materials and Methods

### 2.1. Study Design

This qualitative study used a phenomenological constructivist approach [27] to inductively identify and understand the ascribed patterns and descriptions of meaning emerging from participants’ experiences, through undergraduate students themselves, as well as through the perspective of subject-matter experts who provide services for this population. The research team’s institutional research ethics board granted ethics approval to conduct this study (Certificate Numbers 19-11-041 and 19-11-048).

### 2.2. Study Setting

This study was conducted at a four-year, English-speaking, public post-secondary institution. This research-intensive university is situated across three campuses in Southwestern Ontario, Canada, and has a population of nearly 27,000 undergraduate students. The ACHA’s most recent iteration of their NCHA-II questionnaire revealed an increase in the percentage of students (*n* = 1235) reporting above average stress at this institution (67.4% (95% CI: 64.7–70.0; CI estimated by Lisnyj et al.) in 2019 [28] compared to 57.7% (n = 807) (95% CI: 54.2–61.1; CI estimated by Lisnyj et al.) in 2013 [29]), making it a suitable setting to explore this research topic.

### 2.3. Sampling and Participant Recruitment Strategies

Convenience sampling was used to recruit undergraduate student participants. Recruitment advertisements were posted at several common, high-traffic student areas across the institution’s main campus. Interested students contacted the lead researcher and were given a letter of information and consent form (Supplementary Material Files A and B) via e-mail. Student participants were provided with a 10.00 CAD hospitality gift card as an honorarium in appreciation of their time. Recruitment continued until data saturation was achieved, a concept indicating incoming data yielded little to no additional information [30], resulting in 14 student participants.

Purposive sampling was used to identify on-campus service providers on the basis of their perceived knowledge and interactions with undergraduate students at this institution (e.g., staff from the student wellness centre, student affairs, student housing services, accessibility services, learning services, and relevant student clubs and organizations). Individuals were invited via e-mail to participate in this research (the e-mail recruitment script used, the letter of information provided, and the consent form given are found in Supplementary Material Files C–E). Data saturation was reached, resulting in 24 stakeholder participants.

### 2.4. Data Collection

Rapport building and informed consent were achieved prior to data collection. Single one-on-one, semi-structured interviews were used to gather data from participants between February and April 2020, and ranged between 20 and 44 min (median: 36 min) in length for student participants and ranged between 30 and 55 min (median: 43 min) in length for stakeholder participants. All interviews were initially scheduled to be in-person at a mutually agreed-upon location at the institution’s main campus. However, the coronavirus (COVID-19) pandemic resulted in some interviews being conducted remotely via a video conference or telephone call. A total of 25 interviews (15 stakeholder and 10 student interviews) were conducted before COVID-19 was officially declared a pandemic; 13 interviews (9 stakeholder and 4 student interviews) were conducted after. All interviews were conducted by the lead researcher, audio-recorded, professionally transcribed verbatim, and field notes were taken to ensure that the data were accurately captured. The lead researcher confirmed the accuracy of all transcriptions against the audio recordings. Pseudonyms were used to anonymize each participant’s identity. Additional socio-demographic information was collected from each participant using an electronic survey at the time of the interview (Table 1 and Table 2). The socio-demographic characteristics of respondents were described using percentages and frequencies.

### 2.5. Data Analysis

Thematic data analysis was undertaken using an iterative process using NVivo (QSR International Pty Ltd. Version 12, Doncaster, Australia). The lead researcher reviewed each transcript repeatedly using a constant comparative approach to better understand the data collected and develop codes [31,32,33]. Similar codes were organized and condensed into broader categories to help identify overarching themes and patterns pertinent to this study’s research questions. Participants’ quotations were extracted to support each emerging theme. Socio-demographic data from the electronic survey were imported into Microsoft Excel and analyzed using descriptive statistics.

Consistent with best practices for qualitative thematic analysis, credibility, transferability, dependability, and confirmability were attained through various techniques. These included purposive sampling, prolonged engagement, bracketing, researcher reflexivity, thick description, low-inference descriptors, audio-recorded data, constant comparison, member checking, peer debriefing, triangulation, and maintaining an audit trail [34,35,36].

## 3. Results

Participants identified several principal factors and sub-factors at each level of the socio-ecological model influencing undergraduate students’ academic stress (Table 3).

### 3.1. Factors at the Individual Level

#### 3.1.1. Students’ General Health Status

Large differences exist among student and stakeholder participants’ perceptions of stress. Students predominantly conceptualized stress in a negative context, where it inhibits their capacity. Students described the adverse physical, cognitive, and emotional symptoms that they experience when stressed—specific examples included having a rapid heart rate, stomach pain, feeling nauseous, inability to focus, and feeling restless—whereas most on-campus service provider participants recognized stress exists along a continuum, in which there are negative (i.e., distress) and positive components (i.e., eustress). Both groups conceptualized academic success similarly, describing the importance of academic performance, retention, academic engagement, and academic satisfaction. Both groups of participants acknowledged an inverse relationship between stress and academic success, whereby increased stress is associated with decreased academic success. Both sets of participants agreed on the difficulty of determining causality among these two constructs.

Both sets of participants noted catching a cold or the flu and experiencing a medical emergency as the most common poor physical health outcomes that increase stress and decrease academic success. Students and service providers indicated having generalized anxiety disorder, social anxiety, depression, and panic disorder as the most frequent and severe psychological health factors increasing students’ stress and decreasing their academic success. Many stakeholders identified students struggling with a mental health issue and not acknowledging it as an additional stressor. Both groups of participants indicated that students who engage in health-diminishing behaviours, such as binge drinking, self-medicating, poor sleep hygiene, physical inactivity, and social media overuse, experienced increased stress and reduced academic success, whereas both participants stated students who attend to their physical, mental, and emotional health and well-being by exhibiting health-promoting habits, including having regular physical activity, restful sleep, and practicing self-care, had lower stress and improved academic performance. Students and stakeholders indicated exhibiting adverse characteristics, such as feeling hopeless, burnout, negative self-talk, and imposter syndrome, were detrimental to academic success.

#### 3.1.2. Transition to Post-Secondary Life

The transition to post-secondary life was a key topic discussed by all participants and comprises factors such as experiencing homesickness due to being uprooted from one’s home, family, and childhood friends, adapting to a new campus and city environment, having a higher workload, and having greater control and more responsibilities in one’s life. Both groups of participants also indicated students who feel uncertainty about their lives as another large source of stress. Examples include being unsure what course material will be included on an assessment, being uncertain about their post-graduate career prospects, and feeling each decision being made is consequential to their academic and life trajectory. One student described this drastic change:

*You’re making these huge decisions for the first time. When you’re in high school and living at home, all these choices are being made for you. And now you have a choice, so it’s really scary, and that really stressed me out a lot. (Participant 10)*

This transition also brings several competing demands, including students preparing for course assessments, having volunteer commitments, having home responsibilities, participating in extracurricular activities, being a varsity athlete, having a part-time job, and job shadowing, all while experiencing personal life events. One student clearly demonstrated how these demands influence their stress and academic success:

*Sometimes it’s so hard wearing all my hats as a student. Work, for me, it’s not really a choice. I have to work to pay rent and to ultimately survive. And I’m involved with a lot of clubs and organizations. I see them as investments towards my future and what I want to pursue because, you know, it’ll add to my experience and make my resume as expansive as possible. So, all these things together increase stress, where I usually end up neglecting certain assignments that obviously has an impact on my academic success. (Participant 18)*

A staff member echoed this assertion:

*Students are going to be graduating with hundreds of other students with an undergraduate degree. So, what’s going to make them stand out? Is it an involvement? Is it their extracurriculars? Is it volunteering? This is what inspires them to get involved on campus. So, students want to be competitive, and in doing so, they might overexert themselves. So how do we ensure that students balance their work and life and personal things, while not compromising both their self-care and their values? It all comes down to students’ ability to manage being a full-time student, while also working part-time, while also being a student leader, but also volunteering and also making time for their friends, family, and partner. All these obviously affects how much time and effort they can put towards focusing on their studies. (Participant 11)*

#### 3.1.3. Coping Mechanisms

All participants acknowledged the importance of students having adaptive skills to navigate their transition to post-secondary life successfully. Both groups identified having high levels of self-efficacy, motivation, communication skills, time management skills, and stress management skills as essential for undergraduate students to support their school-life balance. Specific examples mitigating the effects of stress on students’ academics studies include setting and meeting personal and academic goals, breaking tasks down into manageable components, perceiving oneself to control their time, developing a routine, and expressing oneself when experiencing hardships. However, many interviewees indicated that undergraduate students often have inadequate organizational and communication skills and exhibit low academic self-efficacy levels, thereby increasing their stress and threatening their academic success. Examples include students not attending lectures, ruminating on poor grades, expecting one’s instructor to structure and manage their time as in high school, underestimating how much time is required to complete a task, not recognizing the quantity of time spent does not always equate to quality of time spent, engaging in avoidance or distraction coping mechanisms, not meeting self-expectations, and not being able to communicate what they need.

Several student participants further acknowledged their lack of knowledge of navigating institutional information surrounding stress management and academic well-being. Students discussed their challenges in identifying and accessing practical, social, emotional, and health resources across campus, as depicted by the following interviewee:

*I always find myself scrambling to figure all this stuff out for myself, and it takes so long to figure out what the resources are and what certain things even exist. (Participant 17)*

Many stakeholders’ perspectives counter this statement by alluding to undergraduate students’ lack of accountability, where they expect others to hand them everything. One participant commented:

*There’s a certain level of responsibility the University puts on the student, “You are an adult.” We understand our policies are not perfect and it’s not always easy. We understand you have a lot of things going on. We want to help you, but you have to help yourself first. There’s a certain level of responsibility as a student to show up and help yourself. (Participant 5)*

In fact, several on-campus service providers specifically indicated that students lack resiliency skills in responding to their stress. Stakeholders cited helicopter parenting and not experiencing failure in early life as primary reasons contributing to this inadequacy. Two participants succinctly describe this experience as follows:

*Students are used to having someone else solve all their problems for them. It doesn’t matter whether it’s their teacher or their parent or their big brother. So then when they get here, they don’t have the resilience to be able to, and the grit, to be able to deal with those things themselves. (Participant 6)*

*We are seeing an increasing number of students who are coming in lack skills in adversity or in being challenged. I often say to students, “The first time you fail something should not be at university.” (Participant 26)*

Most staff interviewed indicated that the students they interact with generally seek out help in a reactive way once they become too overwhelmed to attenuate the debilitative effects of stress. The following two excerpts demonstrate this situation:

*A big thing I see is many students coming in are more reactive rather than proactive about what they’re needing both academically, as well as socially or in that transition to university. So, it comes from resiliency, like how do you bounce back when things don’t go well? And I think a big individual source of stress for students is they’re here now, and they don’t know how to bounce back from those adversities they’re experiencing at university. There’s a lack of resiliency that’s occurring. (Participant 2)*

*A lot of the students I see, it’s very reactive to what’s going on at that time. They suddenly realize, “Oh, I’ve made some maybe poor choices. I haven’t been thinking about my health. I haven’t been thinking about these things.” So, they’re stressed. So, they’re reacting in that way. (Participant 5)*

#### 3.1.4. Socio-Demographic Factors

Participants noted many socio-demographic factors as a final category that influenced undergraduate students’ stress and academic success. Factors mentioned include age, gender, living arrangements, parental income, parental academic achievement, race, socio-economic status, food security, and financial security. Both groups of interviewees indicated various vulnerable student sub-populations that face additional stressors throughout their educational experience. This includes LGBTQ+ students, ethnic minorities (e.g., Black, Indigenous, and People of Colour (BIPOC) students), students with intersecting identities, students with lived experience of mental illness, mature students, international students, first-generation students, low socio-economic students, students dealing with physical or sexual assaults trauma, and students with a learning, physical, or developmental disability. Stakeholder participants additionally noted minority students experiencing actual or perceived discrimination in their lives. One on-service campus provider concisely summarized the vast experiences of the student population:

*All of the life circumstances that you can imagine, somebody out there on campus is experiencing it. (Participant 9)*

### 3.2. Factors at the Interpersonal Level

#### 3.2.1. Social Support

Participants highlighted various perceived and received tangible and intangible social support types in students’ lives, including emotional, informational, and companionship supports. Greater accessibility, availability, and quality of these supports facilitate a stronger sense of belonging among students, alleviating undergraduates’ stress levels and improving their academic success.

One on-campus service provider participant described the integral role of social support in being academically successful:

*I think social connections are the most important thing when it comes to being successful as a student. (Participant 6)*

Social support is typically derived through various sources in students’ personal lives, including family members, friends, romantic partners, co-workers, pets, health professionals, and clergy. Social support often comes from classmates, roommates, friends inside one’s program of study, academic staff, teaching assistants, instructors, and academic clubs in individuals’ academic lives as a student. One stakeholder participant indicated that the difficulty in determining the most optimal source of social support:

*I think it’s more about who you’re connecting with, so I don’t know that I’d categorize it. I wouldn’t say that friends are better than family or vice versa. It’s more about what the quality of the support is. I connect with students estranged from their families, but they have a fantastic friend group, and that’s amazing. I also have folks very connected with their family and friends, but none of those are positive relationships. (Participant 14)*

Both groups of participants specifically noted that students experience negative social support in their personal life during a break up, when they are unable to navigate a long-distance relationship, and during a conflict with a friend or family member. Both groups also mentioned this same finding when students experience homesickness and friend-sickness, when they are feeling socially isolated or lonely, when experiencing a change in family dynamics (e.g., severe illness or death of a parent, becoming a caregiver, parents divorcing, and having a child), and when they are unable to meet parental expectations. Stakeholder participants additionally mentioned physical, emotional, and sexual violence as less frequently experienced student stressors. Many participants acknowledged that international students face additional negative social factors, including cultural differences in receiving mental health supports, time differences impacting communication with individuals back home, and being physically distant from their support circles. Both sets of participants noted that students experience negative social support in their student life arising from living arrangement difficulties (e.g., social tension with roommates and problems with their landlord), group member conflicts (e.g., unbalanced workloads), and a lack of clear guidance from their instructor or academic staff.

#### 3.2.2. University Culture

Most participants indicated that the university culture presents unique opportunities for individuals to connect, form relationships, and improve their overall student experience. Positive components that decrease stress and enhance academic success include building a social community among like-minded individuals with similar aspirations, learning from one another (e.g., forming study groups), engaging in healthy behaviours (e.g., going to the gym together and referring friends to seek professional help if needed), volunteering, and participating in professional, academic, and social campus events together. Both groups of participants also acknowledge university culture’s adverse implications on students’ stress levels and academic success. Specific examples include building off each other’s stress and spiraling, engaging in normalized unhealthy behaviours (e.g., pulling all-nighters, binge drinking, and consuming energy-dense, nutrient-poor foods), feeling the pressure to fit in and socialize, feeling judged by others, feeling expected to take a full course load (i.e., five half-courses per semester for most undergraduate students), constantly comparing oneself to others (e.g., relationships, grades, and physical appearance), and the academic competitiveness among peers. One stakeholder captured this complexity:

*I think there’s this social stress of fitting in on-campus and finding a group of friends. So, they’re struggling with what is appropriate for them to be doing at this age and what they need to do to fit in? I think they have a false sense of what everyone’s doing, because if you look at the data, students aren’t actually drinking, smoking weed, and having sex as much as they think. Still, they think that everybody participates in this, and so I think it causes stress because they think they’re somehow outside of the norm. (Participant 20)*

### 3.3. Factors at the Institutional Level

#### 3.3.1. On-Campus Programs, Services, and Resources

Participants noted several mental health promotion and mental illness prevention supports on campus that equip students with resources to manage their stress and optimize their academic success. These programs and services are predominantly offered through both the institution’s student affairs and library services. Student affairs at this institution provide services related to athletics, childcare, career advising, student housing, the administration of centres to support specific campus communities (e.g., Indigenous students), health and wellness, and accessibility. Academic program counsellors are another source of information on campus mentioned by many participants.

On-campus resources include information surrounding common predictors of stress, the awareness of signs of distress, establishing effective studying habits, setting and meeting goals, the importance of sleep hygiene, and developing a routine. Undergraduate students are presented with such resources across campus using a combination of active and passive program deliveries. Examples of active programming include students engaging in learning and writing workshops, partaking in stress-buster activities (e.g., therapy dogs and the Stress Less for Tests program), and seeking out services on campus (e.g., individual and group counselling). Examples of passive programming include reading stress-related information posted on the institution’s online learning management system, receiving a handout of available resources during orientation week, seeing a wellness booth setup in the student centre, and listening to mental health resources presented during lecture. One stakeholder noted:

*We do our best to provide a universal design to our programming workshops and sessions. We do our best to provide a universal designed curriculum in everything we do so that, from our perspective, no students or student groups are neglected. We always strive to provide services that consider the diverse needs of students. (Participant 11)*

The importance of tailoring on-campus programs to diverse populations resonated with another stakeholder participant:

*There’s a whole team of us, professional staff, on the university’s campus that support students from other identities. Those services and supports need to exist for those who need it because they’re different supports than the general supports offered to the general student population, whether they’re culturally relevant or culturally responsive. There are different needs across students’ different identities, and multiple identities and intersecting identities, of course. So, having those supports available for the students who need them, when they need them, is essential. However, it’s challenging to have every service custom-tailored to every student from every lived experience. (Participant 32)*

Despite the vast resources available, nearly all participants acknowledged the difficulty of navigating available resources. Both groups of participants specifically demonstrated their frustration with a non-existent central repository or database detailing available programs and services, reaching broken links on institutional webpages (i.e., receiving 404 error codes), and finding inconsistent information across different webpages. One key stakeholder noted this barrier as an opportunity to improve services:

*[This institution] is very rich with resources in terms of where people can go to find support or develop skills. I think that in itself speaks to how that can be overwhelming for students. The overwhelming amount of resources available can sometimes make it difficult to navigate. We need to take a moment to take an inventory of what resources exist, how they’re promoted, how different areas can work together in a more collaborative way to reach students, so it doesn’t feel so siloed. So, like, almost consolidating and bringing them together so that it’s not so overwhelming for students. (Participant 28)*

Participants mentioned additional gaps in on-campus service provision, including programs largely targeting first-year, on-campus students, ignoring the needs of upper-year and off-campus students. Many student participants indicated that their experience with unsuitable programs (e.g., scheduling information sessions on Friday nights), a lack of available multicultural counsellors, extensive wait times to see a counsellor, limited hours of service operation that often conflicted with students’ course schedules, and concerns about the effectiveness of counselling, including uncertainty surrounding what information would not be kept confidential. Many stakeholder participants noted a lack of formal evaluation in services delivered, precarious employment of core program staff making it difficult to know who to refer students to, a lack of suitable services for international students, having limited upstream, proactive efforts to promote mental health, and creating programs reactively once the institution’s reputation was at stake.

Both participant groups agreed on the importance of capturing students’ perspectives in mental health program development to address their needs and preferences. This includes engaging with student clubs and organizations, conducting campus-wide needs assessment surveys, and creating opportunities to establish and maintain communication channels to enhance, extend, and empower the student voice in decision-making processes. The following on-campus service providing participant recognized the value of student input in supporting their goals:

*It’s important to work with the experts on campus, but also chat with students themselves, about what they’d like to see done, like, how can we work best with them to understand how to navigate all the changes in this different environment that they’re experiencing. I think, for the most part, students are pretty good at being able to say, “This is what I’m looking for.” (Participant 19)*

#### 3.3.2. Policies and Procedures

Participants identified numerous processes conducive to supporting student mental health and academic learning. Both sets of participants acknowledged the institution’s commitment to promoting a safe campus environment, including policies and procedures surrounding sexual violence, human rights, accessibility, and equity and inclusion. Service providing participants additionally referred to the institution’s student mental health strategy and embedding a “healthy campus initiative” to strengthen student mental health and well-being. Specific examples mentioned include reading week, deferred examination privileges, not having three exams during a 24 h period, the ability to drop a course until the last class day of the semester, the existence of an undergraduate academic calendar outlining all policies and regulations, an institutional work-study program to assist students with financial need, and the availability of scholarships and bursaries.

Participants also expressed various concerns in existing policies and procedures that elevate undergraduates’ stress. Both groups of participants mentioned the rising costs of pursuing post-secondary education (including some degrees being costlier than others), some instructors requesting a medical note for accommodations, instructors not being required to have mental health training, and difficulty completing scholarship applications. Other institutional gaps mentioned by student participants included having a mandatory on-campus meal plan for students living in select residences, not having time to read the undergraduate academic calendar, and requiring at least a cumulative academic average of 70% to be involved in specific programs (e.g., peer helping). Stakeholder participants additionally noted little guidance surrounding the “healthy campus initiative” and the absence of a student mental health monitoring system. One stakeholder acknowledged the interconnection of such factors at the institutional level:

*For some students, it’s the school causing them stress. So, why would they get the school to help them with their stress, when they’re the reason? Like, I often wonder if that’s part of it. I wonder if that dissuades them from seeking help here because they see being in university as the cause of their stress. (Participant 32)*

Some participants experienced frustrations feeling that the institution was not prioritizing student wellness:

*I see from internal workings the University is treated like a business. I see that effort is put into the donations to the business school or veterinary school, so the courtyard can be redone. And they got, like, 1.8-million dollars for that. Meanwhile, I find it very hard to understand how the University is saying, “Oh, we really care.” We, as a wellness organization, try to advocate and get more funds, or more programming, but we aren’t seeing anything come back to us. So, no, I’m not necessarily convinced that the University is putting wellness above other priorities. (Participant 7)*

*There’s a disconnect between the decision-makers upstairs and the frontline workers who deal with students daily. There’s a disconnect in terms of what decisions are being made and how are we moving forward? The most frustrating part about this whole thing is that they don’t see it. They deal with the dollar signs and we’re dealing with the student emotions. And now you want to increase students’ tuition. But you also say that mental health is a priority for students, but you’re indirectly hurting every single student by making this decision. (Participant 15)*

#### 3.3.3. Undergraduate Courses

This sub-theme was majorly informed by student participants and only rarely mentioned by a few stakeholder participants. Students indicated that their experiences with courses taken, which reduced their stress levels and facilitated their academic success, depended on their instructor. Specific factors included their instructor’s ability to convey course material effectively, making an effort to know their students, encouraging active participation during lecture, encouraging students to attend office hours, offering extensions to students when requested, providing timely grades and constructive feedback, building flexibility into their teaching plans (e.g., reviewing material when not understood and offering alternative modes of assessment to students), dropping students’ lowest marks, providing previous examples of examinations, creating suitable tests, encouraging their own performance feedback, and pointing students to mental health and learning resources. Almost all participants described a perceived power differential between professors and undergraduate students that elevated students’ academic stress.

Both groups of participants further acknowledged the influence of students’ program of study in diminishing their stress levels and supporting their academic journey. Examples mentioned include creating a semester schedule outlining deadlines across different courses, and preparing students beyond graduation (e.g., holding networking events, alumni nights, and resilience skill-building workshops). Student participants described diverse experiences concerning course delivery modes and instructional strategies, such as synchronous and asynchronous learning, online courses, student-led learning, and problem-based learning.

Student participants also mentioned many factors that heightened their stress and threatened their academic success. Examples included having several exams and deadlines during a confined period (e.g., increased evaluation density preceding and following reading week), having steady deadlines, receiving severe grading penalties for late assignments, assessments being heavily weighted, taking courses known to be notoriously difficult (e.g., organic chemistry), having to self-teach all course material to oneself, not being provided a rubric for an assignment, being tested on material not reflective of what was taught, and having closed-book assessments. One student participant insightfully captured this:

*Examination assessments are always inherently more stressful to me because even if you know the course material, there’s this illusion that knowledge only exists in a vacuum within two hours when you don’t have physical access to your course notes, whereas, in the real world, that’s not necessarily how it works. So, I think that’s stressful because it’s not like supernatural. I understand why it needs to exist, but it does put more of an impetus on also memorization rather than understanding. (Participant 37)*

All participants asserted the overwhelming weight students place on their grades being the sole indicator of their academic success. One key informant stakeholder specifically noted:

*We’re all going to need to contribute to society. One of the things that frustrates me is this notion that a university’s prestige and reputation is derived from the cut-off grades of its incoming students, that we’re a more prestigious institution if the cut-off grade average is 92% to get into our program. To me, that’s a mark of shame. That says that you’re not actually here to serve a transformative purpose in the lives of people. You’re here to serve the people who already know how to achieve and will achieve and be successful despite anything you can provide for them. The real mark of success is being able to help somebody who isn’t hardwired to excel in the learning culture that we’ve created and go out and contribute and feel whole and successful in contributing to society. We have to live with 100% of the people who are born in this world. Not just pick out the top 8%. (Participant 24)*

#### 3.3.4. Built Environment

Both participant groups referenced various components of the institution’s built environment that positively influence student health and academic outcomes. On campus, wellness and learning facilities exist, healthy food options are available, green spaces are abundant, walking paths are accessible, bus stops are in convenient locations, bike racks encourage bicycling, buildings are clean, residences support student living, and internet connections are strong and reliable.

Student and stakeholder participants also mentioned deterrents in institutional places and spaces that negatively affect student outcomes, such as exacerbating student stress and adversely impacting their academic success. Factors include unaffordable healthy food choices, restricted time or access to buildings on campus, limited parking spaces, overcrowded study spaces, poor lighting in some buildings, inconsistent signage for gender-neutral bathrooms, a lack of visual representation of diverse cultures throughout campus (e.g., few murals and artwork of non-Caucasian cultures), and an absence of physical centres for many minority populations. One stakeholder participant captured how a lack of representation on campus might diminish one’s sense of belonging:

*I think when you walk into certain spaces, they’re very Western, they’re very colonial. We’re a Western education system. We are a predominantly white institution. We’re still a predominantly white, predominately middle, upper-middle class institution. So, there’s a lot of privilege on our campus. And a lot of students not seeing themselves represented for sure. Um, and that makes it hard. (Participant 32)*

Many participants also spoke about how classroom setups impact student’s learning. Participants agreed that smaller classroom sizes, the use of a whiteboard or chalkboard, and having classroom furniture that is moveable collectively facilitate active learning, while large lecture halls (e.g., auditoriums) with a heavy reliance on slideshows support passive learning. Both participant groups agreed that writing a final examination in a location similar to their classroom alleviated academic stress.

### 3.4. Factors at the Community Level

#### Community Resources

Participants affirmed that the city where the institution’s main campus is situated presents numerous opportunities for supporting undergraduate students’ mental health and academic success. Several community organizations exist to provide aid to students, including a public library, a local hospital, the HIV/AIDS Research and Community Health (ARCH) clinic, a crisis text message line provided by Kids Help Phone, and addictions, mental health, and crisis services offered through Here 24/7 and a Canadian Mental Health Association (CMHA) branch. Participants noted that undergraduate students frequented various facilities throughout the community, often with other students, to alleviate stress. Examples mentioned supporting student gatherings include going to restaurants, coffee shops, the cinema, clubs, bars, recreational centres, and the mall. Participants acknowledged that several community events and activities exist, although it was questioned how many students truly attend them. Both participant groups additionally spoke about the city being incredibly environmentally sustainable, with a wealth of green spaces, trails, and clean water. One stakeholder participant professed:

*I think [city name] does a really, really good job about promoting a healthy, safe environment. [City name] is a very clean city. It’s very welcoming. The people are very friendly here. If I were a parent, I would definitely trust my child going to [institution name] because I know it is in a safe city. And, it’s a city that’s easy to navigate. (Participant 1)*

Two students further corroborated the community’s nurturing feel:

*[City name] provides many opportunities and has many facilities for students to go to in case they’re stressed. (Participant 25)*

*This city is a very safe area, a very friendly community kind of a small-town feel in a decent-sized city, so I really enjoy that. (Participant 38)*

Both sets of participants pointed out few obstacles in the institution’s surrounding community that can influence students’ stress levels and academic studies. Factors noted include unreliable public transit (e.g., students being unable to ride the bus due to it being at full capacity or not showing up at the scheduled time), a lack of available off-campus living options, and expensive housing rental costs.

### 3.5. Factors at the Public Policy Level

Participants only focused on negative factors that adversely impact students’ stress levels and their ability to succeed academically.

#### 3.5.1. Changes to Government Post-Secondary Aid Programs

Changes to the provincial financial assistance program resulted in eliminating free tuition for low-income students and eliminating the interest-free grace period on loans that students need to repay, in addition to a large number of students no longer qualifying for government aid due to reductions in family income thresholds, thereby exacerbating financial concerns among many students. Stakeholder participants also noted that the provincial government’s former Student Choice Initiative (SCI), which was active between January and November 2019, allowed students to select what non-mandatory ancillary fees they would like to opt-out of paying. This limited the capacity of running some on-campus programs and services, as they were receiving significantly less funding. One stakeholder noted that over $500,000 CAD was not collected due to the SCI.

#### 3.5.2. Canadian Federal and Provincial Government Laws and Regulations

International students must abide by the laws set by Immigration, Refugees, and Citizenship Canada (IRCC), a Government of Canada department, when studying at the institution. One stakeholder participant argued that this information is presented in very technical, legal terms often not clearly understood by many international students, making their transition to Canadian post-secondary life even more difficult. Many participants noted insufficient funding from the government surrounding mental health resources, resulting in extreme wait times, understaffed services, and students having unmet mental health needs. Participants further expressed concerns regarding the rising costs of pursuing post-secondary education, in which some degree programs are more expensive than others (e.g., Bachelor of Commerce and Bachelor of Engineering) due to the provincial government deregulating fees. Other participants also voiced concerns surrounding the uncertainty about how future laws may affect their future endeavours (Participant 25 is a student and Participant 19 is a stakeholder):

*When the government creates new laws, I kind of get scared, like, “Okay, how is this going to affect my future?” They’re constantly cutting stuff from the medical field, like hospitals, so it’s going to be harder to get jobs later on, there’s going to be less opportunity. They’re even changing things at schools, so maybe my education won’t be affected, but my kid’s education might be affected, or my grandkid’s education. (Participant 25)*

*I think if you’re in teacher’s college, there’s a lot of stress with the current administration provincially, in terms of decisions that are being made, and how that might affect one’s career goals. And does that now mean students have to look at a teaching career out of province or internationally? What if they don’t want to do that? So, I think that’s certainly a source of stress for students who want a plan but might not know what their life might look like. (Participant 19)*

#### 3.5.3. Uncertainty Surrounding the Coronavirus (COVID-19) Pandemic

Of the 13 interviews (nine stakeholder and four student interviews) completed after the declaration of the COVID-19 pandemic, participants expressed extreme immediate concerns following a lack of guidance from government officials surrounding what the remainder of the semester might look like due to provincial guidelines forcing institutions to physically close, how to continue operating essential services for students, and what financial challenges might arise for post-secondary students and the institution.

## 4. Discussion

This exploratory study aimed to identify the contextual factors influencing the relationship between undergraduate students’ sources of stress and their academic success, through the perspective of undergraduate students and on-campus service providers at a post-secondary institution in Southwestern Ontario. This research served as a needs assessment to determine how aligned students’ perceptions of stress and academic success are compared to staff who provide services to this population within institutions, with the aim of identifying opportunities for future practice and research implications concerning mental health promotion.

The socio-ecological model for health promotion was useful in categorizing our study’s findings to demonstrate the complex interplay of demographic, psychological, emotional, social, physical, and academic factors impacting students’ academic stress. Both groups of participants noted a multitude of factors at each level of the socio-ecological model, with a stronger focus on identifying adverse factors, suggesting that stress is predominantly perceived negatively among undergraduate students. Both sets of participants also identified a higher concentration of factors at the first three socio-ecological model levels (i.e., individual, interpersonal, and institutional). However, our results suggest a trickle-down effect, where some factors at higher levels (i.e., community and public policy) influence other factors at the lower levels of this model. For instance, changes to the provincial financial assistance program at the public policy level, coupled with difficulties in completing scholarship applications at the institutional level, place financial pressures on many undergraduate students to work part time while completing their studies, thereby introducing competing interests that reduce students’ time at the individual level, which can all collectively increase their stress and decrease their academic success. Students belonging to equity-seeking groups face additional stressors at all levels of the socio-ecological model that further exacerbate their stress and impact their academic success. Our results further reveal that certain sources of stress remain constant throughout one’s post-secondary experience (e.g., financial problems, relationship difficulties, and feeling a sense of ambiguity). In contrast, other sources of stress change by academic year (e.g., transitioning to post-secondary life reflects first-year students’ struggles, while concerns surrounding preparing applications for post-graduate programs or jobs are experienced among upper-year students). These findings support Linden and Stuart’s [20] work which identified the most severe and frequent stressors experienced by post-secondary students. In recognizing the complexity surrounding the depth and breadth of factors influencing undergraduate students’ stress levels and academic success at each socio-ecological model level, it is imperative to acknowledge that a simple solution, such as an individual program, will likely not suffice in addressing stress on campus. Post-secondary institutions instead ought to offer a suite of programs, resources, and services on campus, that are known and accessible, and intertwine and intersect across the different levels of the socio-ecological model to create a web of interventions to address this complex issue. It is also imperative for post-secondary institutions to communicate and disseminate this information to students, such as through a centralized repository, to help students navigate available services and resources across campus.

A lack of communication and education appears to underlie many of the identified factors influencing undergraduate students’ stress levels and academic needs, especially at the first three levels of the socio-ecological model. A knowledge mobilization approach [37] can help strengthen communication and educational tactics surrounding mental and academic wellness to maximize reach, relevance, relationships, and ultimately results across the broader post-secondary community audiences. At the individual level, this includes students being unable to identify they are struggling with a mental health problem, being unable to express themselves when experiencing a hardship, constantly feeling ambiguity, and being unable to navigate institutional information surrounding stress management and academic well-being. At the interpersonal level, factors encompass disputes with family or friends due to poor communication and receiving little guidance from course instructors. At the institutional level, factors include a lack of a central repository to navigate existing programs and services, on-campus programs ignoring the needs of upper-year and off-campus students, perceptions surrounding the institution not prioritizing student wellness, and finding it challenging to digest institutional policies and procedures clearly and concisely. It is critical for campuses to have robust communication mechanisms in place, via multiple formats and channels, to promote the awareness, accessibility, and availability of services and programs [38]. Strengthening communication strategies can additionally improve coordination and collaboration among services to better address students’ needs. Institutions must be clear and deliberative in their messaging that promoting mental health and academic success is a shared responsibility among all post-secondary community members [39]. This joint effort includes administration governing comprehensive policies and procedures, staff offering evidence-informed programs and services that optimize students’ needs, faculty members being equipped with appropriate resources to assist students, and students themselves being able to communicate when experiencing hardships. Moving forward, it is also imperative to continue capturing the student voice in refining mental health promotion programs to ensure their evolving needs and preferences are met.

There were minor differences among student and on-campus service provider participants’ responses, where both groups largely identified the same factors at each socio-ecological model level. This finding supports the growing literature recognizing the integral role of post-secondary institutions in fostering students’ optimal mental wellness and learning [16,38]. Both groups of participants indicated that undergraduate students typically seek services reactively to address their mental health concerns once they are already stressed, indicating a gap in proactive, resilience-focused, upstream efforts at post-secondary institutions to prevent the deleterious effects of stress from initially occurring. Programs adopting a positive psychology framework, which emphasizes cultivating students’ strengths and resources, have been found to improve their performances and well-being across several life domains [40,41]. This focus is often absent at post-secondary institutions, calling for further research to deepen the understanding of these approaches and provide targeted proactive mental health interventions across campus, to help diminish stress and improve academic success [11].

### Limitations, and Future Research Directions

This study captured undergraduate students and on-campus service providers’ perceptions concerning the factors affecting the relationship between students’ sources of stress and academic success. This research used convenience sampling, where only registered undergraduate students who were physically present at the institution during the recruitment period would have had the opportunity to see the advertisements to participate. This presents a selection bias in which students who dropped out due to overwhelming stress would not have had the opportunity to contribute to this study’s findings. There may have also been a social desirability bias among participants’ responses, as there was little mention surrounding provocative factors concerning academic stress. There were fewer student participants than on-campus service providers included in this research. While we reached a range of students regarding academic year and study program during recruitment, student participants predominantly identified as female (78.9%), and no international students were recruited. Future research should also consider faculty members as additional participants to gain a more comprehensive understanding of all dominant actors’ perspectives surrounding this topic. This study only interviewed participants at one post-secondary institution, so future studies should be conducted across Canadian campuses to better represent all students across the country. Although this study’s results suggest an inverse relationship between stress and academic success (i.e., as stress increases, academic success decreases), we could not draw any causal inferences about the true direction surrounding this relationship. Future research should develop indicators for all identified factors and utilize a longitudinal study design to reveal causal relationships [42]. A final suggestion for future work to consider is to specifically look at the effects of the COVID-19 pandemic on students’ mental health and academic learning.

## 5. Conclusions

This qualitative study used the socio-ecological model of health promotion to identify factors that influence the relationship among undergraduate students’ stress levels and academic success, through the viewpoints of undergraduate students and staff who provide services for this population. This study’s findings indicate that several overlapping factors exist across the five socio-ecological model levels, demonstrating that stress is complex, with various sources impacting various levels. This research also highlights that it is imperative for post-secondary institutions to establish effective communication channels to educate all relevant stakeholders, including administration, faculty, staff, and students, on the awareness, accessibility, and availability of existing programs and services. Moving forward, this research encourages post-secondary programs to be proactive in developing resiliency skills among the undergraduate student population to help minimize their stress levels and optimize their academic success.

## Figures and Tables

**Table 1:** Undergraduate Student Participants’ Demographic Information and Self-Reported Stress Levels (*n* = 14).

Student Demographics	*n* (%)
Gender Identity	
Female	11 (78.6)
Male	2 (14.3)
Non-binary	1 (7.1)
Academic year	
First-year undergraduate	3 (21.4)
Second-year undergraduate	0 (0.0)
Third-year undergraduate	4 (28.6)
Fourth-year undergraduate	5 (35.7)
Fifth-year undergraduate	2 (14.3)
Enrollment status	
Full time	13 (92.9)
Part time	1 (7.1)
Student status	
Domestic student	14 (100.0)
International student	0 (0.0)
Living arrangement	
Off campus	11 (78.6)
On campus	2 (14.3)
Parent or guardian’s house	1 (7.1)
Cumulative grade point average	
50–59%	1 (7.1)
60–69%	1 (7.1)
70–79%	3 (21.4)
80–89%	6 (42.9)
90–100%	3 (21.4)
Average level of stress during the academic year (1 = extremely low; 10 = extremely high)	
1–5	0 (0.0)
6	1 (7.1)
7	2 (14.3)
8	7 (50.0)
9	2 (14.3)
10	2 (14.3)
Average level of stress in one’s daily life (1 = extremely low; 10 = extremely high)	
1	0 (0.0)
2	0 (0.0)
3	1 (7.1)
4	2 (14.3)
5	3 (21.4)
6	0 (0.0)
7	7 (50.0)
8	1 (7.1)
9	0 (0.0)
10	0 (0.0)
Previous utilization of stress reduction resources from one’s institution	
No	1 (7.1)
Yes	13 (92.9)

**Table 2:** Stakeholder Participants’ Demographic Information and Perceptions of Student Stress Levels (*n* = 24).

Stakeholder Demographics	*n* (%)
Gender identity	
Female	17 (70.8)
Male	5 (20.8)
Non-binary	1 (4.2)
Chose not to respond	1 (4.2)
Appointment status	
Full time	21 (87.5)
Part time	1 (4.2)
Chose not to respond	2 (8.3)
Number of years in current role	
1–4 years	16 (66.7)
5–9 years	4 (16.7)
10–14 years	2 (8.3)
≥15 years	2 (8.3)
Direct contact with students in current role	
No	1 (4.2)
Yes	23 (95.8)
Supervise staff with direct contact with students in current role	
No	6 (25.0)
Yes	18 (75.0)
Level of knowledge related to student stress (1 = extremely low; 10 = extremely high)	
1–5	0 (0.0)
6	2 (8.3)
7	2 (8.3)
8	3 (12.5)
9	12 (50.0)
10	5 (20.8)
Chose not to respond	0 (0.0)
Average level of stress among students during the academic year (1 = extremely low; 10 = extremely high)	
1–5	0 (0.0)
6	1 (4.2)
7	6 (25.0)
8	11 (45.8)
9	3 (12.5)
10	0 (0.0)
Chose not to respond	3 (12.5)
Average level of stress among students in their daily life (1 = extremely low; 10 = extremely high)	
1–3	0 (0.0)
4	3 (12.5)
5	1 (4.2)
6	5 (20.8)
7	9 (37.5)
8	2 (8.3)
9	1 (4.2)
10	0 (0.0)
Chose not to respond	3 (12.5)

**Table 3:** Factors Influencing Undergraduate Students’ Stress Levels and Academic Success Using the Socio-Ecological Model.

Socio-Ecological Model Level	Principal Factor	Sub-Factor
**Individual Level**	Students’ general health status	Perceptions and symptoms of stress Physical health status Psychological health status Health behaviours Attitudinal characteristics
	Transition to post-secondary life	Homesickness Adapting to a new campus and city environment Increased demands and responsibilities Feeling ambiguity
	Coping mechanisms	Human capital and psychological capital Accountability or lack thereof Being reactive rather than proactive
	Socio-demographic factors	Age, gender, living arrangements, parental income, parental academic achievement, race, socio-economic status, food security, and financial security Vulnerable student sub-populations (e.g., ethnic, cultural, and religious minorities)
**Interpersonal Level**	Social support	Types of social support (emotional, informational, and companionship supports) Sources of social support in students’ personal and academic lives Impacts of positive and poor social support
	University culture	Sense of community Positive and negative influences by peers
**Institutional Level**	On-campus programs, services, and resources	Available supports across campus Active and passive modes of delivery Gaps in service provision Importance of capturing the student voice
	Policies and procedures	Regulations concerning student mental health, academic learning, and finances Healthy campus initiative Perceptions of the institution not prioritizing student wellness
	Undergraduate courses	Positive and negative experiences with courses and course instructors Large emphasis placed on grade point average
	Built environment	Physical resources on campus Classroom setup
**Community Level**	Community resources	Diverse mental health and academic supports throughout the community Community events and activities in various sectors Local community strengths (e.g., nurturing and environmentally sustainable feel) Drawbacks in the local community (e.g., poor transit, expensive housing, few living options)
**Public Policy Level**	Financial implications	Changes to government post-secondary aid programs Insufficient funding surrounding mental health resources Rising cost of post-secondary education
	Uncertainty surrounding the coronavirus pandemic	Lack of immediate guidance from government officials

## Data Availability

The data presented in this study are available on request from the corresponding author.

## Supplementary Materials

The following are available online at https://www.mdpi.com/article/10.3390/ijerph18073779/s1, Supplementary File A: Student Participant’s Letter of Information, Supplementary File B: Student Participant’s Consent Form, Supplementary File C: On-Campus Service Provider Participant’s E-Mail Recruitment Script, Supplementary File D: On-Campus Service Provider Participant’s Letter of Information, Supplementary File E: On-Campus Service Provider Participant’s Consent Form.

## References

[1] American College Health Association (2013). American College Health Association-National College Health Assessment II: Canadian Reference Group Data Report Spring 2013.

[2] American College Health Association (2019). American College Health Association-National College Health Assessment II: Canadian Reference Group Data Report Spring 2019.

[3] Wiens K., Bhattarai A., Dores A., Pedram P., Williams J.V., Bulloch A.G., Patten S.B. (2020). Mental health among Canadian postsecondary students: A mental health crisis?. Can. J. Psychiatry.

[4] Reavley N., Jorm A.F. (2010). Prevention and early intervention to improve mental health in higher education students: A review. Early Interv. Psychiatry.

[5] Arnett J.J. (2006). Emerging Adulthood: The Winding Road from the Late Teens through the Twenties.

[6] Eisenberg D., Gollust S.E., Golberstein E., Hefner J.L. (2007). Prevalence and correlates of depression, anxiety, and suicidality among university students. Am. J. Orthopsychiatry.

[7] Zaidlin G., Lisnyj K.T., Dougherty B., Cook N., Papadopoulos A. (2020). Utilizing the Health Belief Model to move post-secondary students toward flourishing mental health. J. Posit. Psychol..

[8] Holinka C. (2015). Stress, emotional intelligence, and life satisfaction in college students. Coll. Stud. J..

[9] Miczo N., Miczo L.A., Johnson M. (2006). Parental support, perceived stress, and illness related variables among first-year college students. J. Fam. Commun..

[10] Frazier P., Gabriel A., Merians A., Lust K. (2019). Understanding stress as an impediment to academic performance. J. Am. Coll. Health.

[11] Lisnyj K.T., Gillani N., Pearl D.L., McWhirter J.E., Papadopoulos A. (2021). Factors associated with stress impacting academic success among post-secondary students: A systematic review. J. Am. Coll. Health.

[12] Eisenberg D., Golberstein E., Hunt J.B. (2009). Mental health and academic success in college. BE J. Econ. Anal. Policy.

[13] Linden B., Stuart H. (2020). Post-secondary stress and mental well-being: A scoping review of the academic literature. Can. J. Community Ment. Health.

[14] Hurst C.S., Baranik L.E., Daniel F. (2013). College student stressors: A review of the qualitative research. Stress Health.

[15] Schonfeld I.S., Farrell E., Rossi A.M., Quick J.C., Perrewé P.L. (2009). Qualitative and quantitative methods in occupational-stress research. Stress and Quality Working Life: The Positive and the Negative.

[16] Lisnyj K.T., Russell R., Papadopoulos A. (2020). Risk and protective factors for anxiety impacting academic performance in post-secondary students. Can. J. High. Educ..

[17] Goodday S.M., Rivera D., Foran H., King N., Milanovic M., Keown-Stoneman C.D.G., Horrocks J., Tetzlaff E., Bowie C.R., Pickett W. (2019). U-Flourish university students well-being and academic success longitudinal study: A study protocol. BMJ Open.

[18] Faulkner G., Ramanathan S., Kwan M. (2019). Developing a coordinated Canadian post-secondary surveillance system: A Delphi survey to identify measurement priorities for the Canadian Campus Wellbeing Survey (CCWS). BMC Public Health.

[19] Linden B. (2019). Development and Psychometric Evaluation of the Post-Secondary Student Stressors Index (PSSI). Doctoral Dissertation.

[20] Linden B., Stuart H. (2019). Psychometric assessment of the Post-Secondary Student Stressors Index (PSSI). BMC Public Health.

[21] Glanz K., Rimer B.K., Viswanath K. (2015). Health Behavior and Health Education: Theory, Research, and Practice.

[22] McLeroy K.R., Bibeau D., Steckler A., Glanz K. (1988). An ecological perspective on health promotion programs. Health Educ. Q..

[23] Orpana H., Vachon J., Dykxhoorn J., McRae L., Jayaraman G. (2016). Monitoring positive mental health and its determinants in Canada: The development of the Positive Mental Health Surveillance Indicator Framework. Health Promot. Chronic. Dis. Prev. Can..

[24] Halladay J., Bennett K., Weist M., Boyle M., Manion I., Campo M., Georgiades K. (2020). Teacher-student relationships and mental health help seeking behaviors among elementary and secondary students in Ontario Canada. J. Sch. Psychol..

[25] Chen J., Bloodworth R., Novak P., Le Cook B., Goldman H.H., Rendall M.S., Thomas S.B., Reynolds C.F. (2018). Reducing preventable hospitalization and disparity: Association with local health department mental health promotion activities. Am. J. Prev. Med..

[26] Castillo E.G., Ijadi-Maghsoodi R., Shadravan S., Moore E., Mensah M.O., Docherty M., Aguilera Nunez M.G., Barcelo N., Goodsmith N., Halpin L.E. (2019). Community interventions to promote mental health and social equity. Curr. Psychiatry Rep..

[27] Van Manen M., Adams C., Baker E., Peterson P., McGaw B. (2010). Qualitative research: Phenomenology. International Encyclopedia of Education.

[28] American College Health Association (2013). American College Health Association-National College Health Assessment II: University of Guelph Executive Summary Spring 2013.

[29] American College Health Association (2019). American College Health Association-National College Health Assessment II: University of Guelph Executive Summary Spring 2019.

[30] Glaser B.G., Strauss A.L. (1967). The Discovery of Grounded Theory: Strategies for Qualitative Research.

[31] Miles M.B., Huberman A.M., Saldaña J. (2014). Qualitative Data Analysis: A Methods Sourcebook.

[32] Creswell J.W., Hanson W.E., Clark Plano V.L., Morales A. (2007). Qualitative research designs: Selection and implementation. Couns. Psychol..

[33] David M., Sutton C.D. (2011). Social Research: An. Introduction.

[34] Baxter J., Eyles J. (1997). Evaluating qualitative research in social geography: Establishing ‘rigour’ in interview analysis. Trans. Inst. Br. Geogr..

[35] Lincoln Y.S., Guba E.G. (1985). Naturalistic Inquiry.

[36] Russell C.K., Gregory D.M., Cullum N., Ciliska D., Haynes R.B., Marks S. (2008). Evaluation of qualitative research studies. Evidence-Based Nursing: An Introduction.

[37] Squires V. (2019). Voices and insights: Using student voice to understand and address mental health issues on campus. Papers on Postsecondary Learning and Teaching, Proceedings of the University of Calgary Conference on Learning and Teaching, Calgary, Canada, 30 April–1 May 2019.

[38] Canadian Association of College & University Student Services and Canadian Mental Health Association https://healthycampuses.ca/wp-content/uploads/2014/09/The-National-Guide.pdf.

[39] Research Impact Canada. http://researchimpact.ca/so-what-the-heck-is-knowledge-mobilization-and-why-should-i-care.

[40] Carmona-Halty M., Salanova M., Llorens S., Schaufeli W.B. (2019). Linking positive emotions and academic performance: The mediated role of academic psychological capital and academic engagement. Curr. Psychol..

[41] Luthans B.C., Luthans K.W., Jensen S.M. (2012). The impact of business school students’ psychological capital on academic performance. J. Educ. Bus..

[42] Martínez I.M., Youssef-Morgan C.M., Chambel M.J., Marques-Pinto A. (2019). Antecedents of academic performance of university students: Academic engagement and psychological capital resources. Educ. Psychol..

