# Supplementary material for: Exploration of Factors Affecting Post-Secondary Students’ Stress and Academic Success: Application of the Socio-Ecological Model for Health Promotion
Source: Int J Environ Res Public Health. 2021 Apr 5;18(7):3779. doi: 10.3390/ijerph18073779 (PMC8038589; doi:10.3390/ijerph18073779)
Supplement: Supplementary file 1 [file ijerph-18-03779-s001.zip › Supplementary File E.pdf]

CONSENT (INTERVIEW PARTICIPANT)

Exploring On-Campus Service Providers' Perceptions of Stress Impacting Post-Secondary Students' Academic Success

- I agree to participate in the study ***Exploring On-Campus Service Providers' Perceptions of Stress Impacting Post-Secondary Students' Academic Success***.
- I have read the information presented in the information letter about a study being conducted by Konrad Lisnyj, a graduate student in the Department of Population Medicine at the University of Guelph.
- I have had the opportunity to ask questions about my involvement in this study and to receive additional details I requested.
- I understand that personally identifying information will not be used as data or published within the report.
- I understand that the one-on-one, semi-structured interview will be audio-recorded and the data I provide may be hand-written.
- I understand that if I agree to participate in this study, I may withdraw from the study at any time or up until May 1st, 2020. If I choose to withdraw, all of my data will be destroyed.
- I have been given a signed copy of this form.

Please answer the following questions:

(1) I agree that the interview can be audio recorded.
a) Yes
b) No

(2) I agree that the researcher can take notes during the interview.
a) Yes
b) No

(3) I would like to receive a summary of the study's results.
a) Yes
b) No

If yes, where would you like the results sent:

____
E-mail address

____
Mailing address

____

____
Name of Participant (Printed) Signature Date

Consent form explained in person by:

____
Name and Role (Printed) Signature Date
