# Supplementary material for: Exploration of Factors Affecting Post-Secondary Students’ Stress and Academic Success: Application of the Socio-Ecological Model for Health Promotion
Source: Int J Environ Res Public Health. 2021 Apr 5;18(7):3779. doi: 10.3390/ijerph18073779 (PMC8038589; doi:10.3390/ijerph18073779)
Supplement: Supplementary file 1 [file ijerph-18-03779-s001.zip › Supplementary File A.pdf]

UNIVERSITY of GUELPH

IMPROVE LIFE.

LETTER OF INFORMATION

Exploring Sources of Stress Impacting Post-Secondary Students' Academic Success

Investigators:

Principal Investigator:
Dr. Andrew Papadopoulos
Department of Population Medicine
University of Guelph
Guelph, ON, Canada
apapadop@uoguelph.ca

Student Investigator:
Konrad Lisnyj, PhD Candidate
Department of Population Medicine
University of Guelph
Guelph, ON, Canada
klisnyj@uoguelph.ca

Purpose of the Study

You are invited to take part in a qualitative study exploring post-secondary students' perceptions about sources of stress impacting academic success. I am conducting this research study to fulfill the partial thesis requirements for my Doctor of Philosophy in Population Medicine degree.

This project seeks to better understand the contextual factors that promote or prevent stress in your academic career, as well as to identify any gaps in current service provision at the University of Guelph (UofG). This information will help guide future research and practice at the UofG to prevent stress from unduly impacting students' academic success.

Procedures Involved in the Research

The semi-structured interview will be one-on-one and face-to-face, and will take between 30-45 minutes to complete. It will take place at your most convenient time on the UofG campus between February and April 2020. With your permission, I would like to use an audio recorder and take handwritten notes during the interview to ensure the data collected accurately represents your experiences and insights surrounding this topic. As a participant, you have the right to refuse to answer any question that you find uncomfortable or do not wish to for any reason, or you can end the discussion at any time. All audio recordings and notes taken will be destroyed following the completion of this project. Until then, they will be password-protected and saved under an alias on my encrypted computer.

The interview will begin with myself providing a brief overview of what the purpose of this project is. This will be followed by asking you general questions about your perceptions of stress and academic success. For example, "What comes to mind when you think of the term "stress"? and "How do you conceptualize academic success?" You will also be asked about the common sources of stress you experience in your life as a student and what things you do/can do to mitigate stress. The purpose of such questions is to better understand the breadth and depth of the factors influencing academic stress, as well as to identify opportunities to improve research and program provision here at the UofG. This will be concluded with asking you questions about the barriers you perceive/experience that dissuade students from seeking help from the University, as well as asking you questions about how you would like the University to better support student stress mitigation.

Potential Harms, Risks or Discomforts

The risks, harms, and discomforts involved in participating in this study are minimal. However, there is a possibility some topics or themes brought up during the interview may cause you to feel uncomfortable or uneasy if you recount difficult experiences. You will be provided with information about on-campus counselling services, and you will be referred to appropriate counselling resources should you become distressed.

However, it is important to recognize you do not need to answer any questions or discuss any topics that may make you feel uncomfortable. As well, you can stop the interview at any time, even after you begin the interview or withdraw from the study once the interview is over.

Potential Benefits

Although the research may not benefit you directly, it provides you with an excellent opportunity to share your experiences and insights about the specific factors that you believe increase or decrease student academic stress. In turn, this can help the University of Guelph better understand how students experience stress and address any gaps in service provision. This can help inform the planning and development of a stress mitigation intervention at the University.

Payment or Reimbursement

In appreciation of your time, you will receive a $10.00 University of Guelph hospitality gift card.

Confidentiality

You are participating in this study confidentially. I will not use your name or any information that would allow you to be identified by others. No one but me will know whether you participated in this study unless you choose to tell them. This is also an important factor to consider when selecting your desired date, time, and location on campus to conduct this interview to ensure your participation remains confidential if you so choose to participate.

Identifying information will be replaced with pseudonyms in interview transcripts and study notes. Data without identifying information will be shared with my supervisory committee. The information you provide will be summarized with the information provided by other participants. The information you provide will be kept on a computer that will be protected by a password and saved under your pseudonym to ensure anonymity. Once the study has been fully completed, all of the data collected from you, as well as the other participants, will be deleted.

Participation and Withdrawal

Your participation in this study is voluntary. It is your choice to be part of the study or not. If you decide to be part of the study, you can decide to stop, at any time, even after signing the consent form or part-way through the study. If you decide to withdraw, there will be no consequences for you. In cases of withdrawal, any data you have provided will be destroyed unless you indicate otherwise. You can choose not to answer some questions, yet still be in the study. If you choose to withdraw from the study, you will still receive the $10.00 UofG hospitality gift card.

You can no longer withdraw from this study after May 1st, 2020, when I expect to begin analyzing the data collected and preparing my manuscript for submission. Please also note that you do not waive any legal rights by agreeing to take part in this study.

Information About the Study Results

I expect to have this study completed by approximately August 2020. If you would like a summary of the results, please let me know how you would like it sent to you.

Questions About the Study

If you have questions or need more information about the study itself, please contact me by email at **klisnyj@uoguelph.ca**.

This study has been reviewed and approved by the Research Ethics Board [REB #19-11-041] for compliance with federal guidelines for research involving human participants. If you have any questions about your rights and welfare as a research participant in this study, please contact: Manager, Research Ethics; University of Guelph; reb@uoguelph.ca; (519) 824-4120 ext. 56606.
