# Supplementary material for: Exploration of Factors Affecting Post-Secondary Students’ Stress and Academic Success: Application of the Socio-Ecological Model for Health Promotion
Source: Int J Environ Res Public Health. 2021 Apr 5;18(7):3779. doi: 10.3390/ijerph18073779 (PMC8038589; doi:10.3390/ijerph18073779)
Supplement: Supplementary file 1 [file ijerph-18-03779-s001.zip › Supplementary File C.pdf]

Dear (name of participant),

I hope this e-mail finds you well!

My name is Konrad Lisnyj, and I am currently a PhD candidate in the Department of Population Medicine at the University of Guelph (UofG) under the supervision of Dr. Andrew Papadopoulos. I am conducting a study to identify sources of stress impacting post-secondary students' academic success through the perspective of staff at the UofG who provide services to students. This project serves as a needs assessment with the goal of creating a stress mitigation intervention, in collaboration with the Student Wellness Centre, to reduce student stress here at the UofG.

As a participant, I am inviting you to partake in a semi-structured, one-on-one, face-to-face interview surrounding this topic at your most convenient time on campus in the coming weeks before the end of April 2020. The interview will be audio-recorded to ensure accuracy and will take between 30 to 45 minutes of your time to complete.

Please be assured there are minimal risks associated with carrying out this study. Your confidentiality will be protected; you will not be identified as taking part in this study in any thesis, presentation or publication that I might produce based on your responses. If you choose to participate, you may decide to withdraw at any time before May 1, 2020, and there will absolutely be no consequences. You will have the opportunity to review the information you have provided after transcription. Furthermore, after the completion of this study, all documents and information collected will be disposed of.

I would also like to assure you that the Research Ethics Board has approved this study for compliance with federal guidelines for research involving human participants #19-11-048.

I would like to thank you in advance for your time and consideration. If you are interested in participating in this research project, you may contact me at any time by email at klisnyj@uoguelph.ca.

Thank you greatly again for the consideration, and I look forward to hearing back from you!

Sincerely,
Konrad
